# Impact of Competitive Flow on Hemodynamics in Coronary Surgery: Numerical Study of ITA-LAD Model

**DOI:** 10.1155/2012/356187

**Published:** 2012-09-10

**Authors:** Jinli Ding, Youjun Liu, Feng Wang, Fan Bai

**Affiliations:** College of Life Science and Bioengineering, Beijing University of Technology, Beijing 100124, China

## Abstract

Competitive flow from native coronary artery is considered as a major factor in the failure of the coronary artery bypass grafts. However, the physiological effects are not very clear. The aim is to research the impact of competitive flow caused by different left anterior descending (LAD) artery stenosis degrees on hemodynamics in internal thoracic artery (ITA) bypass graft. An idealized ITA-LAD model was built in CAD tools. The degree of the competitive flow was divided into five classes according to different LAD stenosis degrees: higher (no stenosis), secondary (30% stenosis), reduced (50% stenosis), lower (75% stenosis) and no competitive flow (fully stenosis). Finite volume method was employed for the numerical simulation. The flow velocity distributions, wall shear stress and oscillatory shear index were analyzed. Results showed that higher competitive flow in the bypass graft would produce unbeneficial wall shear stress distribution associating with endothelial dysfunction and subsequent graft failure. The coronary bypass graft surgery was preferred to be carried out when the LAD stenosis was higher than 75%.

## 1. Introduction

In the treatment of coronary artery stenosis, the arterial grafting causes the blood of the bypass graft flowing into the native coronary artery, and competitive flow is often observed near the end-to-side anastomosis region. Several reports provided that competitive flow in the native coronary artery was related to the occlusion or narrowing of the bypass graft supplying this coronary vessel. For example, Pevni et al. suggested competitive flow was the cause for nonfunctioning grafts to vessels with noncritical stenosis (70%), when grafts were occluded or severely stenotic [1]. Nordgaard et al. assessed whether coronary graft flow patterns are affected differently by native coronary competitive flow or by stenosis of the coronary anastomosis based on nine pigs underwent off-pump grafting of the left internal thoracic artery to the left anterior descending artery (LAD). And it was concluded that the mammary graft flow was significantly reduced by native coronary competitive flow, but marginally decreased by a stenotic anastomosis of 75% mean luminal stenosis [2]. Kawamura et al. studied the effect of competitive flow on patency rate of the internal thoracic artery to the left anterior descending artery bypass from the concomitant saphenous vein (SV) graft in the left coronary artery, based on 313 patients who had two bypasses to the left coronary artery including 1 in situ ITA-LAD graft. it was also concluded that competitive flow from SV graft could play an important role in occlusion of the in situ arterial graft [3]. Runwei et al. studied the relation between competitive flow and graft flow in coronary artery bypass grafting based on twelve adult healthy dogs and concluded that competitive flow would restrain the graft flow [4]. The patency of coronary artery bypass grafts (CABGs) is mainly determined by the progression of atherosclerosis and intimal hyperplasia within the grafts as well as technical failures [5]. It has been reported that competitive flow is associated with graft failure, and it is considered to be one of the major factors affecting early arterial graft patency and causing the graft to constrict and fail [6]. Moreover, competitive flow is thought to determine the narrowing of the whole length of the left internal thoracic artery graft, the so-called “string phenomenon” [7]. 

Although, the negative impact of competitive flow on graft patency or failure has been reported in several studies, the exact pathophysiological effects are not fully understood because most of these studies do not provide clear explanations for their findings. Hemodynamics has been widely acknowledged to have significant influence on the arterial diseases. Flow disturbances are confirmed to be related to the intimal hyperplasia and arterial thrombosis [8, 9]. Wall shear stress (WSS) may play an important role in graft patency after CABG [10]. The association between competitive flow and hemodynamics is still unclear. There is rare literature focusing their study on this area, except Qiang et al. who investigated the impact of competitive flow on wall shear stress in mammary artery bypass grafts and confirmed graft flow was highly dependent on the degree of competitive flow [11]. In that paper, competitive flow was simply divided into three degrees and the degree of LAD stenosis was not fully studied. The associations between the degree of LAD stenosis and hemodynamics were not clear.

The aim of the present study is to numerically research the impact of competitive flow on hemodynamics in ITA-LAD model with different degrees of LAD stenosis. This study will give bright understanding of competitive flow and clinical help to surgeons in the treatment of proximal LAD stenosis.

## 2. Method

### 2.1. Model

**Figure 1:**
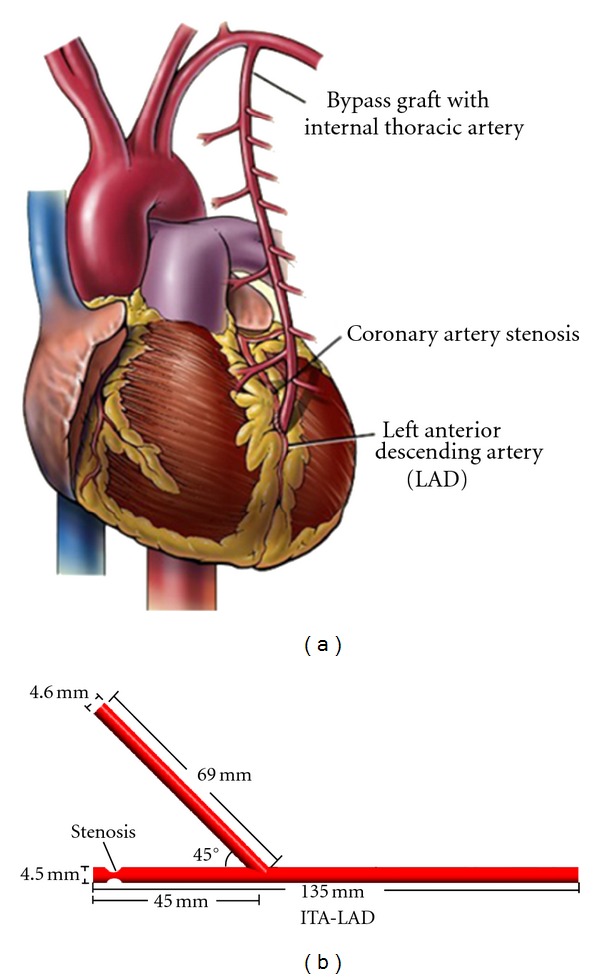
The schematic diagram of ITA-LAD bypass graft (a) and the idealized ITA-LAD model (b).

An idealized coronary artery bypass graft models were built according to the common ITA-LAD bypass design in the treatment of the LAD stenosis (Figure 1). The diameter of the bypass graft was 4.6 mm. The diameter of the native LAD was 4.5 mm [12, 13]. The anastomosis angle of the bypass graft was approximately 45°. The model was imported into the commercial software ANSYS-CFX for meshing. A hexahedral mesh was mainly generated. In order to highlight the hemodynamic features near the wall, the boundary mesh was refined. A mesh sensitivity analysis was carried out using a simple steady computation by comparing the mean pressure and wall shear stress distributions to ensure the accuracy of the simulations. Based on the principle of the acceptable error less than 1%, finally, approximately 1,018,002 grid numbers were generated for the ITA-LAD model.

### 2.2. Computational Fluid Dynamics

**Figure 2:**
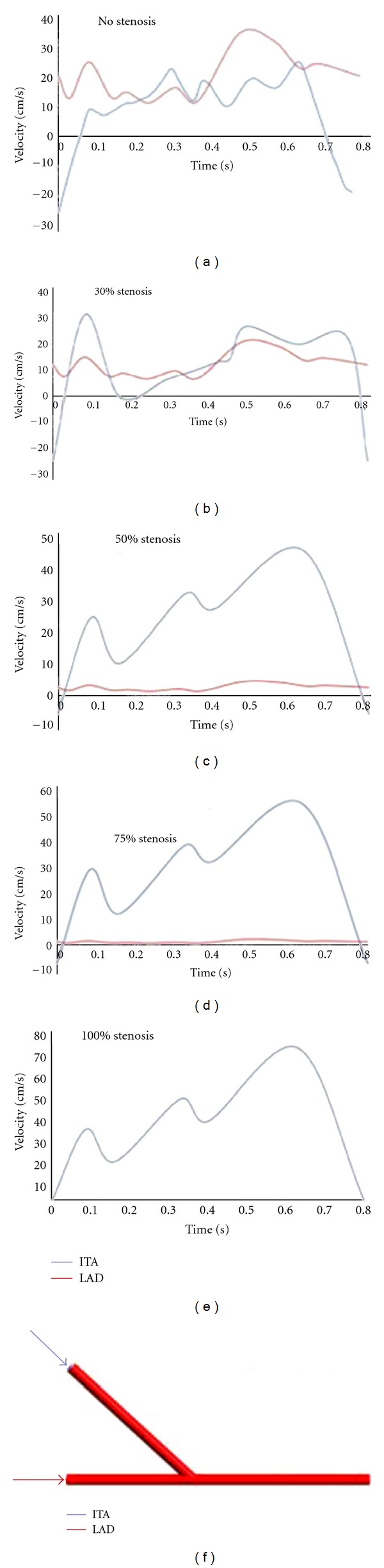
The flow conditions.

Finite volume method was employed for the computational fluid dynamics (CFD). The duration of a cardiac cycle is 0.8 s. The density and dynamic viscosity of blood fluid are 1050 kg/m^3^ and 0.0035 Pa·s, respectively. The blood flow velocity profile of the LAD artery and the bypass graft (Figure 2) were measured from in vivo experiments of eighteen pigs [12, 13]. The degree of the competitive flow in each option was divided into five classes according to different LAD stenosis: higher (no stenosis), secondary (30% stenosis), reduced (50% stenosis), lower (75% stenosis), and no competitive flow (fully stenosis). A relative pressure of zero was set at the outlet. All the arterial walls were assumed to be rigid with the nonslip conditions. The maximum Reynolds number in all the simulations was approximately 1050, and, therefore, laminar flow was studied. The blood flow was assumed as nonsteady, Newtonian and incompressible flow. The adopted cyclic convergence criterion was based on the velocity components and pressure calculations. In addition, a relative error of less than 1*e* − 6 for each calculation was accepted. The Navier-Stokes equations were solved using the ANSYS-CFX package (version 12) on a Microsoft Windows XP 32-bit PC with 4 GB RAM and a dual-core 2.83 GHz CPU. Three cardiac cycles with a fixed time step of 0.001 s were adopted. Comparing the results between the third and the second cardiac cycles, the errors of pressure and wall shear stress were all less than 0.8%. In our opinion, these errors became acceptable, and the results in the third cardiac cycle were used in this study.

### 2.3. Definition of Time-Averaged Wall Shear Stress and Oscillatory Shear Index

Wall shear stress plays an important role in graft patency after CABG [10]. The oscillatory shear index (OSI) is a measure which allows quantifying the change in direction and magnitude of the wall shear stress [10]. And areas of high OSI are predisposed to endothelial dysfunction and atherogenesis [14]. Therefore, time-averaged wall shear stress (TAWSS) and OSI were calculated according to their expressions [11]:

(1) TAWSS=1T∫0T|τ→w|dtOSI=12(1−|∫0Tτ→wdt|∫0T|τ→w|dt),

where ${\vec{\tau }}_{w}$ is the wall shear stress vector, *T* is the time period of the flow cycle, and *τ*_0_ is the wall shear stress for Poiseuille flow at the mean flow Reynolds number. OSI ranges from 0 to 0.5, where 0 describes a total unidirectional wall shear stress and 0.5 means an oscillatory shear flow with a net amount of zero wall shear stress [11].

## 3. Results

### 3.1. Flow Pattern

**Figure 3:**
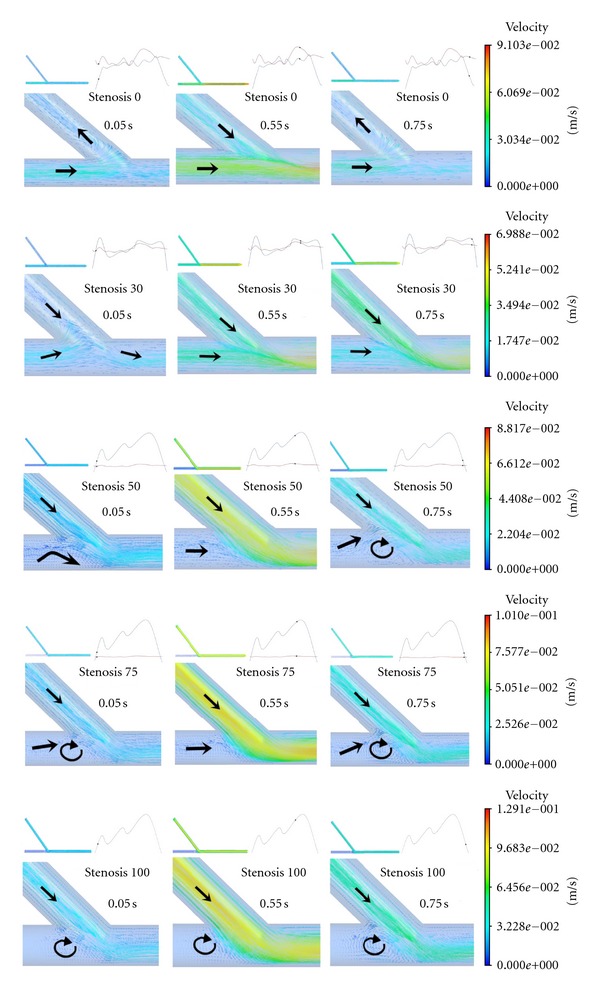
The flow velocity contour maps in the middle section for the nine conditions.

Three typical times including 0.05 s, 0.55 s, and 0.75 s are assigned for the analysis of the flow details. The flow velocity vector maps in the middle section of the five conditions are shown in Figure 3. It is obvious that the flow velocity distributions in the proximal LAD and ITA were mainly influenced by the flow conditions on LAD and ITA inlets, respectively. The higher the degree of LAD stenosis was, the lower the mean velocity in the proximal LAD artery was. And the opposite phenomena were observed in the bypass graft.

At the initial and final periods of the cardiac cycle when the stenosis was lower than 50%, obvious reverse flow was observed in ITA (Figure 2). This would result in lower velocity flow or even reverse flow in the distal LAD artery (Figure 3). However, this phenomenon was hardly observed when the stenosis was higher than 75%. The flow velocity distributions were different according to different inlet conditions, especially near the anastomotic region where disturbed flows, including flow recirculation and flow separation, were observed.

### 3.2. Wall Shear Stress Distributions

**Figure 4:**
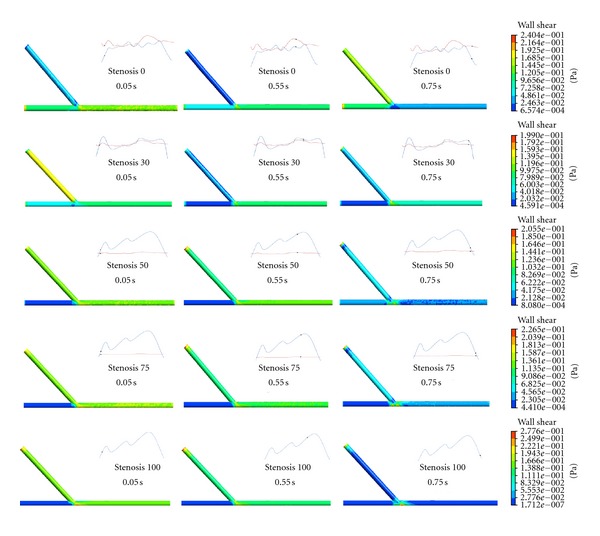
The wall shear stress distributions in the five conditions.

**Figure 5:**
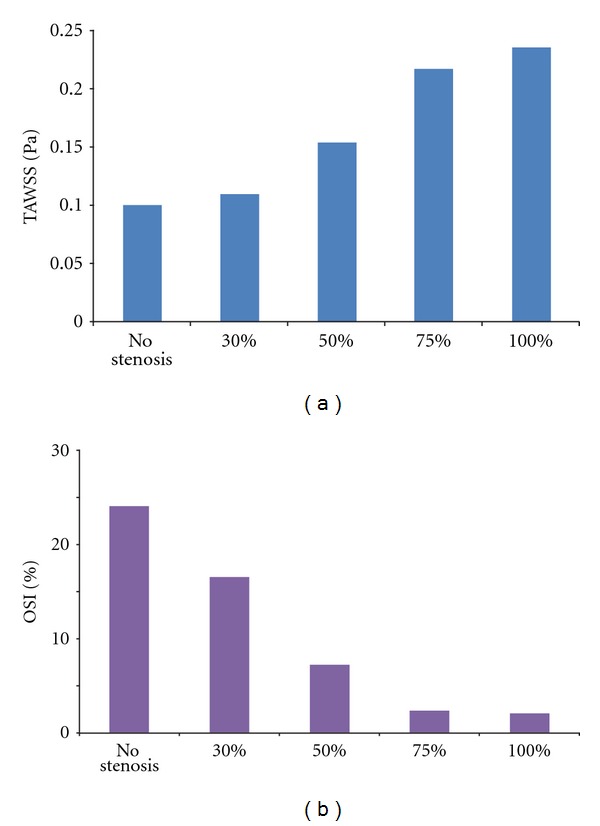
The TAWSS (a) and OSI (b) distributions.

The wall shear stress distributions in the five conditions are shown in Figure 4. Obviously, the highest wall shear stresses are all concentrated on the toe or bed of the anastomosis. The quick variation of wall shear stress mainly appeared near the anastomosis region. The TAWSS and OSI were calculated in the bypass graft, as Figure 5 shows. The TAWSS ranged from 0.1–0.24 Pa, while OSI ranged from 0.02–0.25 for the five conditions.

## 4. Discussions 

The purpose of this study is to investigate the physiological effect of competitive flow on hemodynamics in coronary artery bypass graft. Different degrees of the competitive flow caused by different LAD stenosis were studied in this study.

The flow results shown in Figure 3 revealed that the flow details in the bypass graft were mainly affected by their inlet flow conditions. No disturbed flow was observed inside of each graft except the region near the anastomosis. This may indicate that the degree of competitive flow has significant influence on the flow pattern near the anastomosis. Obvious reverse flow was observed in ITA in the higher, secondary and reduced competitive flow, and this would result in lower velocity flow or even reverse flow in the distal LAD artery. This coincides with the research from Bezon et al. and Berger et al. who reported that the patency rate of the internal thoracic artery to the left anterior descending artery bypass is reduced by competitive flow [15, 16].

The aetiology of graft failure due to competitive flow has not been thoroughly investigated. Wall shear stress plays an important role in graft patency after CABG [10, 17]. Also, areas of high OSI are predisposed to endothelial dysfunction and atherogenesis [14]. Results of our study showed that the TAWSS increased gradually and OSI decreased gradually as the degree of LAD stenosis became higher. Comparing to the TAWSS for no competitive flow, it decreased 7.8%, 34.6%, 53.4%, and 57.3% for that of lower, reduced, secondary, and higher competitive flow, respectively. Comparing to the OSI for no competitive flow, it increased 16%, 102.4%, 685.8%, and 1035.8% for that of lower, reduced, secondary, and higher competitive flow, respectively. It can be seen that the higher competitive flow resulted in the least TAWSS and the OSI in the ITA graft, which indicated that higher competitive flow in the bypass graft would lead to unbeneficial wall shear stress distributions on the bypass graft for low TAWSS and high OSI would increase the probability of intimal hyperplasia and atherogenesis. This might explain why the higher competitive flow would result in the high risk of bypass failure. 

There are also rare researches reporting the clinical associations between CABG and degree of stenosis. Results in this study showed that TAWSS was highest and OSI was lowest for the no competitive flow condition. From the view point of hemodynamics, the probability of intimal hyperplasia and atherogenesis was less when the LAD was fully occluded. Furthermore, the differences of TAWSS and OSI between the lower competitive flow (75% stenosis) condition and no competitive flow (100% stenosis) condition were slight. This indicated that lower competitive flow resulted in TAWSS and OSI similar to the no-competitive flow condition, which agreed well with Nordgaard's reports [2]. Unfortunately, when the stenosis was lower than 75%, the TAWSS decreased and OSI increased fast towards an unbeneficial situation. It might be concluded that, from the comparisons of TAWSS and OSI, the CABG was preferred to be carried out when the LAD stenosis was higher than 75%.

CFD is has been widely used for the recreation of flow fields exiting in idealized or complex geometries of pulsatile flow conditions [18, 19]. The accuracy of a CFD simulation is highly dependent on the quality of geometry and boundary conditions used. However, there are some limitations in this study. The time-dependent pressure conditions were ignored in the CFD simulation. Although the conclusions in this study agreed well with other literatures, it seemed difficult in generalizing our findings to actual one or every patient and describing the detailed hemodynamic features in every coronary bypass anastomosis due to large variation in the anastomotic structure of coronary arteries. Other limitations are related to the CFD models and material assumptions. Firstly, the geometrical models were idealized models rather than the realistic anatomy. This simplification could affect the flow details inside the bypass graft. Secondly, compressible blood, non-Newtonian rheology, and elastic wall were not considered in the CFD simulations as lots of researches did [10, 17, 19]. All these have certain influences on the simulation results and will be considered in the future work. Thirdly, no clinical validation was provided. Future work will dedicate to the clinical experiments and fluid-structure interactions.

## 5. Conclusions

Physiological effect of competitive flow on hemodynamics in coronary artery bypass graft was studied in this paper. Different degrees of the competitive flow caused by different stenosis in the ITA-LAD option were considered. It was concluded that higher competitive flow in the bypass graft would produce unbeneficial wall shear stress distributions consistent with endothelial dysfunction and subsequent graft failure. Lower competitive flow generated higher TAWSS and lower OSI similar to the no-competitive flow condition. The coronary bypass graft surgery was preferred to be carried out when the LAD stenosis was higher than 75%.

## References

[1] Pevni D, Hertz I, Medalion B (2007). Angiographic evidence for reduced graft patency due to competitive flow in composite arterial T-grafts. *The Journal of Thoracic and Cardiovascular Surgery*.

[2] Nordgaard H, Nordhaug D, Kirkeby-Garstad I, Løvstakken L, Vitale N, Haaverstad R (2009). Different graft flow patterns due to competitive flow or stenosis in the coronary anastomosis assessed by transit-time flowmetry in a porcine model. *European Journal of Cardio-Thoracic Surgery*.

[3] Kawamura M, Nakajima H, Kobayashi J (2008). Patency rate of the internal thoracic artery to the left anterior descending artery bypass is reduced by competitive flow from the concomitant saphenous vein graft in the left coronary artery. *European Journal of Cardio-Thoracic Surgery*.

[4] Runwei M, Ruobin W, Huiming G, Shaoyi Z (2005). Relation between competitive flow and graft flow in coronary artery bypass grafting. *Chinese Journal of Clinical Thoracic and Cardiovascular Surgery*.

[5] Nwasokwa ON (1995). Coronary artery bypass graft disease. *Annals of Internal Medicine*.

[6] Sabik JF, Blackstone EH (2008). Coronary artery bypass graft patency and competitive flow. *Journal of the American College of Cardiology*.

[7] Villareal RP, Mathur VS (2000). The string phenomenon. An important cause of internal mammary artery graft failure. *Texas Heart Institute Journal*.

[8] Chiu J, Chien S (2011). Effects of disturbed flow on vascular endothelium: pathophysiological basis and clinical perspectives. *Physiological Reviews*.

[9] John LCH (2009). Biomechanics of coronary artery and bypass graft disease: potential new approaches. *The Annals of Thoracic Surgery*.

[10] Nordgaard H, Swillens A, Nordhaug D (2010). Impact of competitive flow on wall shear stress in coronary surgery: computational fluid dynamics of a LIMA-LAD model. *Cardiovascular Research*.

[11] Qiang F, Yanwen B, Jianmin Y (2006). Effects of competitive blood flow from differently stenotic coronary artery on internal mammary artery graft flow. *Journal of Shandong University*.

[12] Jianmin Y (2006). *The experimental study of the effect of competitive flow and vasoactive drugs on arterial and veinal grafts flow after coronary artery bypass grafting [Ph.D. dissertation]*.

[13] Fukumoto Y, Hiro T, Fujii T (2008). Localized elevation of shear stress is related to coronary plaque rupture. A 3-dimensional intravascular ultrasound study with in-vivo color mapping of shear stress distribution. *Journal of the American College of Cardiology*.

[14] Davies PF (2009). Hemodynamic shear stress and the endothelium in cardiovascular patho-physiology. *Nature Clinical Practice Cardiovascular Medicine*.

[15] Bezon E, Choplain JN, Maguid YA, Aziz AA, Barra JA (2003). Failure of internal thoracic artery grafts: conclusions from coronary angiography mid-term follow-up. *Annals of Thoracic Surgery*.

[16] Berger A, MacCarthy PA, Siebert U (2004). Long-term patency of internal mammary artery bypass grafts: relationship with preoperative severity of the native coronary artery stenosis. *Circulation*.

[17] Vasava P, Jalali P, Dabagh M, Kolari PJ (2012). Finite element modelling of pulsatile blood flow in idealizedModel of human aortic arch: study of hypotension and hypertension. *Computational and Mathematical Methods in Medicine*.

[18] Vignon-Clementel IE, Marsden AL, Feinstein JA (2010). A primer on computational simulation in congenital heart disease for the clinician. *Progress in Pediatric Cardiology*.

[19] Chaichana T, Sun Z, Jewkes J (2012). Computational fluid dynamics analysis of the effect of plaques in the left coronary artery. *Computational and Mathematical Methods in Medicine*.

